# Supplementary material for: Amino Acid Transporters in Plants: Identification and Function
Source: Plants (Basel). 2020 Jul 31;9(8):972. doi: 10.3390/plants9080972 (PMC7466100; doi:10.3390/plants9080972)
Supplement: Supplementary file 1 [file plants-09-00972-s001.zip › Supplementary File/Supplemental Figure S1.docx]

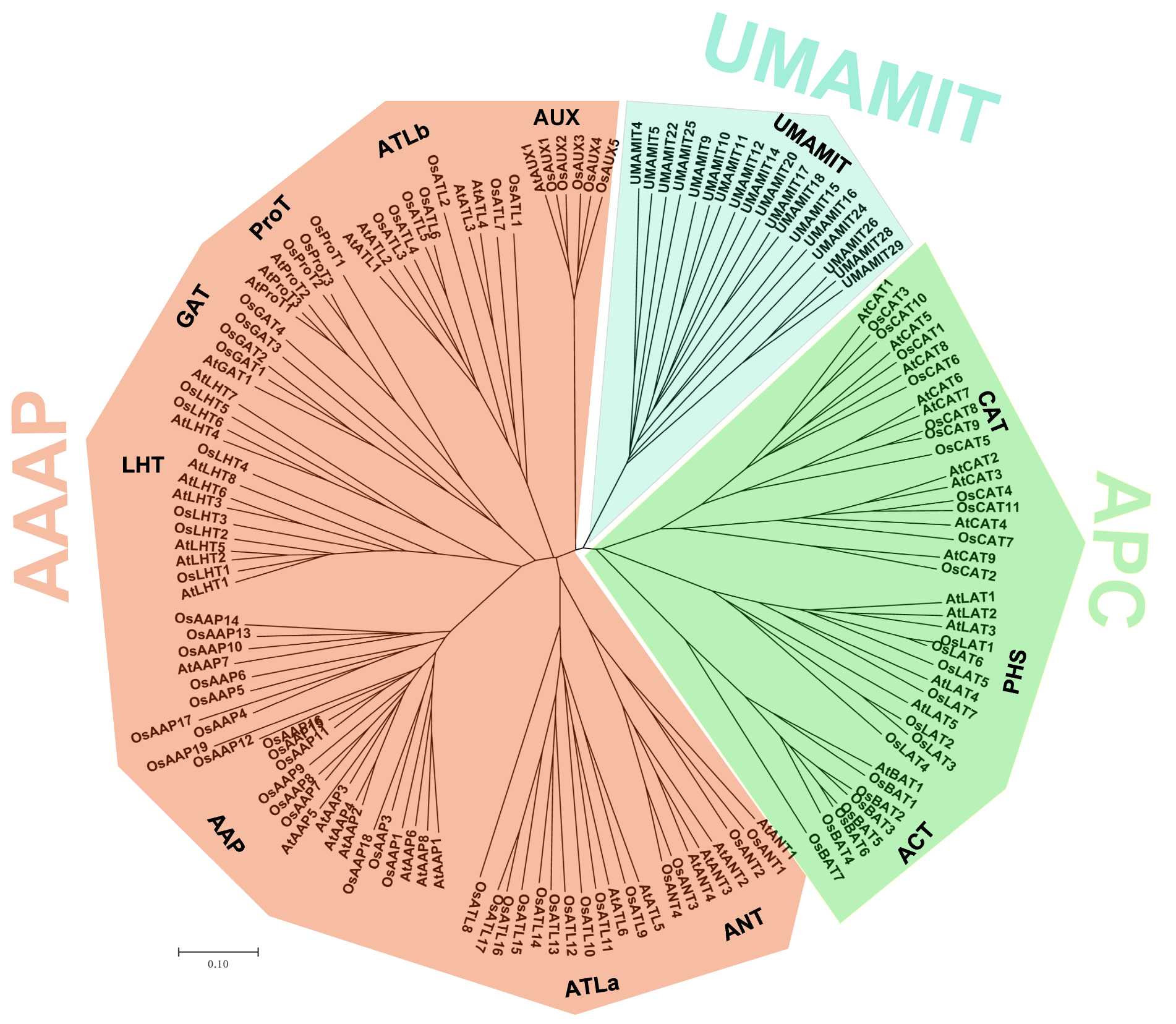


**Supplemental Figure S1**: Phylogenetic relationship of Arabidopsis and rice amino acid transporters. Multiple sequence alignment of full-length proteins was performed by Clustal X and the phylogenetic tree was constructed using MEGA7 with the neighbor-joining method. The tree was divided into 12 subgroups. AAAP, APC and UMAMIT families are marked by different color backgrounds.
